# EBF factors drive expression of multiple classes of target genes governing neuronal development

**DOI:** 10.1186/1749-8104-6-19

**Published:** 2011-04-30

**Authors:** Yangsook S Green, Monica L Vetter

**Affiliations:** 1 Department of Neurobiology and Anatomy, University of Utah School of Medicine, Salt Lake City, UT 84132, USA

## Abstract

**Background:**

Early B cell factor (EBF) family members are transcription factors known to have important roles in several aspects of vertebrate neurogenesis, including commitment, migration and differentiation. Knowledge of how EBF family members contribute to neurogenesis is limited by a lack of detailed understanding of genes that are transcriptionally regulated by these factors.

**Results:**

We performed a microarray screen in *Xenopus *animal caps to search for targets of EBF transcriptional activity, and identified candidate targets with multiple roles, including transcription factors of several classes. We determined that, among the most upregulated candidate genes with expected neuronal functions, most require EBF activity for some or all of their expression, and most have overlapping expression with *ebf *genes. We also found that the candidate target genes that had the most strongly overlapping expression patterns with *ebf *genes were predicted to be direct transcriptional targets of EBF transcriptional activity.

**Conclusions:**

The identification of candidate targets that are transcription factor genes, including *nscl-1*, *emx1 *and *aml1*, improves our understanding of how EBF proteins participate in the hierarchy of transcription control during neuronal development, and suggests novel mechanisms by which EBF activity promotes migration and differentiation. Other candidate targets, including *pcdh8 *and *kcnk5*, expand our knowledge of the types of terminal differentiated neuronal functions that EBF proteins regulate.

## Background

Throughout animal development, many processes must occur coordinately, including patterning, commitment, differentiation and migration of progenitor cells. In the nervous system in particular, these processes are exceedingly complex and depend on the coordinated expression of many sets of genes. A detailed understanding of gene regulation, including knowledge of the hierarchy of transcriptional activity and the types of genes that different transcription factors target, is therefore a critical foundation for understanding nervous system development. One group of transcription factors expressed strongly in the developing nervous system is the early B cell factor (EBF; also called Collier/Olf/Ebf (COE), and Olf/Ebf (O/E)) family of zinc finger helix-loop-helix proteins.

The EBF family includes EBF1, 2, 3 and O/E-4 in mammals [1-6], with EBF2 and EBF3 being the known family members in *Xenopus *[7,8], and ZCOE2 a family member in zebrafish [9]. Invertebrate members of this family include Collier in *Drosophila*, and UNC-3 in *Caenorhabditis elegans *[10,11]. EBF family proteins contain a DNA binding domain (a zinc finger coordination motif), which can also participate in dimerization and transactivation, an atypical helix-loop-helix domain, which is critical for formation of homo- and heterodimers, and a carboxy-terminal domain, which is important for transactivation [2,4,12].

EBF proteins influence multiple processes during development of multiple lineages, including neurons (reviewed in [13,14]). One of their functions is a role in stabilizing neuronal cell commitment. For example, Dubois *et al*. [7] showed that EBF2 can affect neuronal progenitor cell commitment in early *Xenopus *embryos by reinforcing the expression of the proneural basic helix-loop-helix (bHLH) transcription factor NGNR-1, and by maintaining the expression of *delta1*. In addition, in developing chick spinal cord, electroporated mouse *Ebf1 *drives expression of *Ngn1 *and *Ngn2 *[15].

EBF proteins also have critical roles in neuronal cell differentiation. For example, overexpression of *ebf2 *and *ebf3 *leads to ectopic expression of neuronal-specific markers like *n-tubulin *and *nf-m *in *Xenopus *embryos [7,8], suggesting that EBF2 and EBF3 may drive specific aspects of the neuronal differentiation program. Consistent with this, in *Ebf1 *null mouse striatum, early neuronal cells show abnormal expression of several genes, indicating disruption of the process of differentiation [16]. EBF proteins have also been shown to regulate aspects of cell differentiation in both *Drosophila *and *C. elegans *ventral nerve cord [17,18], as well as in early chick spinal cord, where electroporated mouse *Ebf1 *promotes expression of numerous neuronal markers [15].

EBF proteins have been shown to regulate neurite formation and axon guidance, including thalamocortical fibers in the mouse lateral ganglionic eminence [16,19], olfactory axons projecting to the mouse olfactory bulb [20], as well as motor neurons in *C. elegans *[11]. EBF proteins also are critical for neuronal cell migration. For example, EBF factors regulate the migration of gonadotropin releasing hormone (GnRH-1)-synthesizing neurons from the olfactory epithelium to the hypothalamus [21], the migration of Purkinje neurons from the anterior cortical transitory zone to beneath the external granular layer in cerebellar cortex [22], and the migration of facial branchiomotor neurons in the hindbrain [23]. Furthermore, when *Ebf1 *is misexpressed in chick spinal cord, neuroepithelial progenitors migrate toward the mantle layer faster than normal, and the expression levels of *NF *and *R-cadherin *are upregulated [15].

*Ebf *genes are strongly expressed in differentiating central and peripheral neurons throughout development [1,7,8,24], and clearly govern diverse aspects of neuronal development. However, it is not fully understood how these functions are executed since there has not previously been a systematic analysis of EBF transcriptional targets involved in neuronal development. The goals of this study were as follows. First, we performed a microarray analysis to identify candidate targets of EBF3 activity in the developing *Xenopus *nervous system. Second, we analyzed the expression of the candidate targets, to compare their expression with the *ebf *genes and to gain an understanding of where in the embryo they may function. Third, we performed gain- and loss-of-function studies of EBF2 and EBF3 in *Xenopus *embryos to analyze *in vivo *the dependence of the discovered candidate targets on EBF activity, and to confirm the microarray results *in vivo*. Finally, we assessed which candidate target genes are likely to be direct targets of EBF3, and which are indirect targets, to better understand the hierarchy of transcriptional control by EBF proteins. Many genes previously demonstrated to be required for neuronal development are strongly upregulated by EBF, but were not previously known to be targets of EBF transcriptional activity. These targets include transcription factors, cell structural proteins, an ion channel protein, and a gene involved in transforming growth factor (TGF)-beta signaling. The variety of targets found expands our knowledge of the kinds of processes EBF proteins regulate, and reinforces the idea that EBF proteins can influence many aspects of neuronal development because they direct expression of several different functional classes of genes. The discovered candidate targets open a new window to understanding the broader scope of EBF functions.

## Materials and methods

### Generation of hormone-inducible constructs

To generate hormone-inducible constructs, a hormone-binding domain of the human glucocorticoid receptor (hGR) was fused to the coding region of *Xenopus ebf3 *(or *ebf2*), with a myc tag epitope between hGR and the coding region (Additional file 1A) [25,26]. The *ebf3 *coding region was obtained by excision from pBS-Xebf3 [8] with the restriction enzyme DraI, which produces a DNA fragment with blunt ends. The hormone-binding domain was obtained from the pCS2+hGR-MT vector (hGR with myc tag) by cutting with XbaI and treatment with Klenow (Promega, Madison, WI, USA) to generate blunt ends followed by dephosphorylation of the 3' ends with CIP (New England Biolabs, Ipswich, MA, USA). The excised *ebf3 *was then subcloned into the pCS2+hGR-MT vector. To generate hGR-XEBF2 construct, pBS-Xebf2 [8] was used as a template and the following primers containing XhoI and XbaI sites were used to amplify *ebf2*: 5'-GGCC*CTCGAG***ATGGATCCAATCCA**-3' and 5'-GGCC*TCTAGA*T**TCACATGGGCACC**-3' (residues in italics are XhoI and XbaI sites, respectively; bold sequences are the *ebf2 *sequence). The amplified products were digested with XhoI and XbaI and subcloned into the pCS2+hGR-MT vector. Following sequence verification, the *in vivo *expression of these fusion proteins was verified by injecting capped mRNAs into *Xenopus *embryos at the one-cell stage and then performing western blotting with lysates of gastrula stage embryos with 9E10 antibody (Santa Cruz Biotechnology, Santa Cruz, CA, USA), which recognizes the myc epitope (data not shown).

### Microinjection of RNA and morpholinos

The following constructs were used as DNA templates to make capped mRNA: pCS2+Noggin [27], pCS2+hGR [28], pCS2+hGR-MT-Xebf2, pCS2+hGR-MT-Xebf3, pCS2+MT-DN-Xebf, and pCS2+nβgal [29]. Capped mRNA was generated *in vitro *using the Message mMachine kit (Ambion, Austin, TX, USA). Antisense morpholino oligonucleotides (MO) were designed by Gene Tools (Philomath, OR, USA), and directed against a region at or near the translational start site of *ebf2 *(5'-GCGCTTTGTCTCTCAAGGCAGTTCC-3') and *ebf3 *(5'-GTATATTTTCCTGAATCCCAAACAT-3').

For microarray experiments, 1 ng of hGR-XEBF3 mRNA and 0.2 ng Noggin mRNA were co-injected into *Xenopus *embryos at the one-cell stage. Alternatively, 0.4 ng hGR mRNA and 0.2 ng Noggin mRNA were co-injected in control embryos. At stage 9, animal caps were dissected from the embryo, using either a Gastromaster or a hypodermic needle tip. Animal caps were treated with 30 μM dexamethasone (DEX) in 1× Marc's modified Ringer solution (MMR) for 4.5 hours before harvesting of total RNA (Additional file 1B).

For all other microinjections, a volume of 4 nl or 5 nl containing capped mRNA or MOs was injected into one blastomere of two-cell stage embryos in the following amounts: hGR (0.5 ng), hGR-XEBF2 (0.5 ng for overexpression, 0.1 ng for the MO rescue), hGR-XEBF3 (0.5 ng), DN-XEBF (2 ng), MyoD-hGR (0.5 ng), nβgal (30 pg), EBF2 MO (15 ng), EBF3 MO (15 ng) and standard control MO (30 ng). In the MO experiments, both EBF2 MO and EBF3 MO were co-injected. For all injections nβgal capped mRNA was co-injected as a tracer. Embryos were grown until neurula or tail bud stages [30]. hGR-XEBF2, hGR-XEBF3 and MyoD-hGR injected embryos were treated with 30 μM DEX from the gastrula stage (stage 11/11.5) to the neurula stage (stage 14/15). Embryos were then fixed with 4% paraformaldehyde in phosphate-buffered saline for 30 minutes. After washing embryos three times with phosphate-buffered saline, X-gal staining was performed as described [31], followed by post-fixation in 4% paraformaldehyde for 1 hour at room temperature or overnight at 4°C.

### Microarray analysis

Total RNA was isolated from animal caps with the RNeasy mini kit (Qiagen, Germantown, MD, USA). This RNA was used to perform two-color microarray analysis on the *Xenopus *Agilent microarray by the University of Utah Microarray core facility. Fluorescently labeled cRNA, containing either cyanine 3-CTP or cyanine 5-CTP, was generated using the Agilent Two-Color Quick Amp Labeling kit (catalog number 5190-0444). Next, microarray hybridizations were performed using Agilent surehyb hybridization chambers. Slides were then scanned in an Agilent Technologies G2505B microarray scanner at 5 μm resolution. Finally, TIF files were generated from the scanned microarray image, and loaded into Agilent Feature Extraction Software version 9.5.1. LOWESS-normalized data from the Feature Extraction software was filtered to remove control features and features flagged as 'nonuniform'. The LOWESS-normalized intensity values were loaded into Genesifter software (Geospiza, Seattle, WA, USA) for analysis. A *t*-test was applied to data from four biological replicate experiments. *P*-values from the *t*-test were adjusted for multiple testing using the Benjamini and Hochberg method. Genes were selected that showed at least two-fold differential expression and had adjusted *P*-values < 0.05.

### *In situ *hybridization

The following constructs were used to generate antisense RNA probes: pBS-Xebf2 [8], pBS-Xebf3 [8], pBS-Sox2 [32], PCDH8 (IMAGE ID 6955713, ATCC), Peripherin (IMAGE ID 4959167, ATCC), GREB1 (IMAGE ID 5569934, ATCC), pBS-XNF-M [8], KCNK5 (IMAGE ID 6863628, ATCC), NSCL-1 (IMAGE ID 5514274, ATCC), pBS-XNeuroD [33], AML1 (IMAGE ID 4963637, ATCC), Activin beta B (IMAGE ID 5440215, ATCC), EMX1 (IMAGE ID 6957219, ATCC). Antisense RNA probe was generated *in vitro *using SP6, T7 or T3 RNA polymerase (Ambion) and labeled with digoxigenin-11-UTP (Roche, Indianapolis, IN, USA). Whole mount *in situ *hybridization was performed on the fixed and X-gal stained embryos as described [34,35].

### Real-time quantitative PCR

For real-time quantitative PCR (RT-QPCR) experiments, 1 ng hGR-XEBF3 mRNA and 0.2 ng Noggin mRNA were co-injected into *Xenopus *cells at the one-cell stage and animal caps were isolated at stage 9. The animal caps were divided into four groups. The control group received no treatment (-C-D). The second group was treated with 30 μM DEX alone for 3 hours (-C+D), and the third group was treated with 5 μg/ml cycloheximide (CHX) alone for 3.5 hours (+C-D). Finally, the fourth group was treated with 5 μg/ml CHX for 30 minutes and then 30 μM DEX was added for 3 hours (+C+D). Total RNA was purified from animal caps with Trizol (Invitrogen) and then genomic DNAs were removed with the RNeasy mini kit (Qiagen).

To make cDNA from the isolated total RNA from animal caps, the SuperScript III reverse transcriptase (Invitrogen) was used according to the manufacturer's instructions, and then QPCR was performed using Power SYBR Green PCR Master Mix (Applied Biosystems, Carlsbad, CA, USA) on a 7900HT Real Time PCR System (Applied Biosystems). Alternatively, the Superscript III Platinum two step RT-QPCR kit and SYBR Green (Invitrogen) were used to make cDNA and to generate the PCR solution, and QPCR was performed on the same 7900HT Real Time PCR system. MacVector Software was used to design the gene-specific primers (Additional file 2). The relative gene expression level was determined by normalizing the threshold cycle (Ct) of each gene to the Ct of histone H4. One Ct difference indicates a two-fold difference in the initial cDNA template amount. Finally, expression levels were normalized by setting the expression level in the condition of -C+D to 100.

## Results

### Identification of candidate targets of EBF3 in animal caps

**Figure 1:**
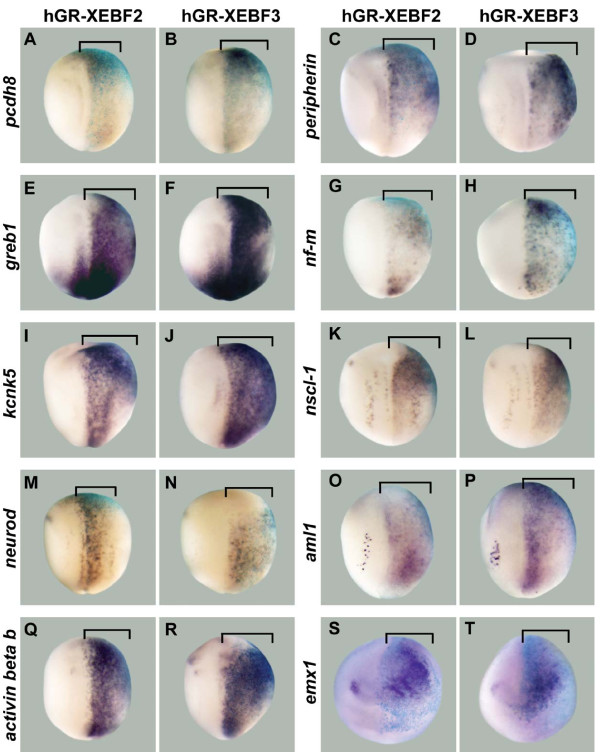
**Candidate target genes upregulated by overexpression of EBF2 or EBF3**. hGR-XEBF2 or hGR-XEBF3 mRNA was injected into one cell of two-cell stage embryos, followed by DEX treatment from the late gastrula stage (stage 11/11.5) to the neurula stage (stage 14/15). β-Galactosidase (β-gal) mRNA was co-injected as a marker of the injected side. In all panels the right side is the injected side, showing the light blue color of X-gal staining. **(A-T) **The expression levels of *pcdh8 *(A,B), *peripherin *(C,D), *greb1 *(E,F), *nf-m *(G,H), *kcnk5 *(I,J), *nscl-1 *(K,L), *neurod *(M,N), *aml1 *(O,P), *activin beta b *(Q,R), and *emx1 *(S,T) are strongly upregulated by EBF2 and EBF3 (brackets). Panels (A-R) show dorsal views, while (S,T) show anterior views.

To identify transcriptional targets of EBF3, we performed a microarray screen comparing the transcripts expressed in *Xenopus *animal cap ectoderm with and without active *Xenopus *EBF3 protein. To control EBF3 activity, we generated a hormone-inducible fusion protein (hGR-XEBF3) that can be regulated by the hormone DEX (Additional file 1A) [26]. We verified that the fusion protein hGR-XEBF3 replicates EBF3 activity *in vivo *by demonstrating that hGR-XEBF3 activated by DEX treatment can induce ectopic expression of *n-tubulin *(data not shown) and *neurofilament-m *(*nf-m*) (Figure 1H), similar to unmodified EBF3 [8]. For all microarrays, we injected mRNA encoding hGR-XEBF3 into one-cell stage embryos, collected animal caps at the blastula stage, and treated with DEX for 4.5 hours (to activate hGR-XEBF3 and induce target gene expression), followed by isolation of total RNA (Additional file 1B). Control animal caps without active hGR-XEBF3 were generated in the same way, but without the addition of DEX.

EBF proteins are involved in the development of B cells, adipocytes, and muscle cells, as well as of neurons (reviewed in [13,14,36]), and the activities of EBF are tissue specific [37]. Since we were interested primarily in neuronal-specific targets of EBF activity, we co-injected embryos with Noggin mRNA to neuralize the animal caps [27]. Agilent *Xenopus *microarrays were used to compare target gene expression levels in DEX-treated animal caps to those in control DEX-untreated animal caps in four independent experiments. To exclude genes that had their expression levels affected by the hormone DEX alone, we performed a separate, control microarray analysis using animal caps treated with DEX expressing control hGR versus untreated animal caps expressing hGR-XEBF3 (Additional file 1B). After removal of the small number of genes that were affected by DEX itself, we found 602 genes that were upregulated more than two-fold, and 504 genes that were downregulated more than two-fold by EBF3 activity (Additional file 3; Gene Expression Omnibus (GEO) accession [GEO:GSE25734]). We found 242 genes upregulated more than five-fold, and 115 genes downregulated more than five-fold.

In cases of incomplete annotation for the *Xenopus *microarray, we used NCBI UniGene or BLAST to identify homologs in other species and determine likely gene identity. The list of candidate targets includes genes with known functions in neurons, as well as genes with established roles in other tissue types, including muscle. Since EBF proteins have not previously been implicated in vertebrate muscle development, an analysis of EBF3 targets with functions in muscle tissue are described in a separate study (YS Green and ML Vetter, submitted). An indication of the integrity of our screen was the strong upregulation of *nf-m*, a known EBF3 target gene [8] (Table 1). As a whole, the results of this array provide an expansive data set for future study of EBF3 activity in *Xenopus*.

**Table 1:** Candidate targets of EBF activity

Gene name	Function	FC^1 ^ (microarray)	FC^2^ (RT-QPCR)	GenBank
*pcdh8*	Transmembrane protein	66	93^a^	BC074360
*nr2f2*	Nuclear receptor TF	47	ND	BC078057
*wnt3a*	Wnt signaling ligand	45	ND	L07538
*peripherin*	Type III intermediate filament	37	32^a^	BC056020
*greb1*	Estrogen-regulated gene	32	21^b^	BC043838
*hoxd10*	Homeodomain TF	31	ND	BC061944
*nf-m*	Type IV intermediate filament	28	29^b^	BC078128
*kcnk5*	K+ ion channel	27	6^b^	BC084931
*nscl-1*	bHLH TF	26	7^b^	BC084434
*neurod*	bHLH TF	26	164^b^	BC072996
*en-2*	Homeodomain TF	24	ND	X62974
*aml1*	Runt-related TF	22	24^a^	BC057739
*activin beta b*	TGF-beta superfamily member	21	154^b^	S61773
*emx1*	Homeodomain TF	16	15^b^	BC077629

The genes chosen for analysis are shown with their known functional roles. FC^1 ^refers to the average fold change in expression by microarray analysis after DEX treatment for 4.5 hours compared to control, in four replicate experiments. FC^2 ^refers to the fold change in expression after DEX treatment for 3 hours compared to control, as detected by RT-QPCR using independent samples (see Figure 6 and Additional file 6 for details). ^a^Performed three replicate experiments; ^b^performed once. FC, fold change; ND, not determined; TF, transcription factor.

### Classes of candidate target genes with predicted neuronal function

The list of targets with predicted neuronal function was very promising, so for further analysis, we selected from the microarray results 14 genes that were more than 10-fold upregulated and that we predicted to be involved in neuronal development, based on expression patterns and functions known from the published literature, as well as on our own observations with whole mount *in situ *hybridization (WM-ISH; discussed below). Although the number of downregulated genes in our array results is comparable to the number upregulated, we focused on upregulated genes for the remaining analysis to identify potential mediators of EBF function in the nervous system. The selected EBF3 candidate target genes were classified based on their known or predicted functions, and these are summarized in Table 1. To confirm the microarray results for these genes, reverse transcriptase PCR (RT-PCR) was performed using independent animal caps from those used in the microarray experiments. For each tested gene, these RT-PCR results showed significant upregulation of gene expression by activated hGR-XEBF3, matching the results found with the microarray analysis (data not shown). As described in detail later, for a subset of the genes we performed RT-QPCR using independent samples, and also found upregulation of gene expression (Table 1). To determine whether neuralization of the animal caps was required for EBF regulation of candidate target genes, we performed an additional microarray experiment using the same conditions, but without Noggin mRNA co-injection (Additional file 4; GEO accession [GEO:GSE27084]). We found that all 14 genes were still upregulated by hGR-XEBF3 in the presence of DEX, suggesting that they can be activated by EBF activity in both neuralized and non-neuralized ectoderm.

After classification, we found that a large number of the selected candidate EBF targets are transcription factors - for example, NR2F2 (nuclear receptor subfamily 2, group F, member 2, also called COUP-TFII) [38], HOXD10 (homeobox D10, also called HOX4D) [39], NSCL-1 (neuronal stem cell leukemia, also called XHEN1 and NHLH1) [40], NeuroD [33], EN-2 (engrailed 2) [41], AML1 (acute myeloid leukemia, also called RUNX1) [42,43], and EMX1 [44]. The fact that we find many transcription factors strongly upregulated by EBF proteins suggests that there are multiple levels of transcriptional control that involve the activity of EBF proteins. For the most part, the remainder of the strongly upregulated candidate targets that we chose are involved in cell structure and neuronal function, including PCDH8 (protocadherin 8) [45], WNT3a [46], Peripherin (also called XIF3) [47] and NF-M (Neurofilament-M) [48], KCNK5 (potassium channel subfamily K member 5, also called TASK2) [49], and Activin beta B (also called INHBB) [50], reinforcing the idea that EBF proteins are involved in neuronal differentiation during development, as well as performing various functions in mature neurons.

### EBF2 and EBF3 are sufficient for the expression of candidate neuronal targets *in vivo*

We previously showed that the protein sequences of *Xenopus *EBF2 and EBF3, as well as their functions in neuronal development in *Xenopus*, are very similar [8]. For these reasons, we included both EBF2 and EBF3 in the experiments that follow. In order to both confirm the microarray data and to determine if EBF2 and EBF3 are sufficient for the expression of the candidate target genes *in vivo*, we examined the expression levels of candidates after overexpression of hGR-XEBF2 and hGR-XEBF3. Overexpression was achieved by injection of mRNA for hGR-XEBF2 or hGR-XEBF3 into one cell of two-cell stage embryos, followed by treatment of the embryos with DEX from the gastrula stage (stage 11/11.5) to the neurula stage (stage 14/15). The expression level of candidate target genes was then examined by WM-ISH. We found that 10 of the 14 candidate target genes were upregulated by overexpression of EBF2 and EBF3 (Figure 1). These were *pcdh8 *(20/23 embryos by EBF2, 27/28 embryos by EBF3), *peripherin *(15/15, 35/35), *greb1 *(genes regulated by estrogen in breast cancer; 12/12, 10/10), *nf-m *(15/15, 35/38), *kcnk5 *(11/11, 39/40), *nscl-1 *(9/9, 33/40), *neurod *(20/21, 39/39), *aml1 *(12/12, 11/11), *activin beta b *(11/11, 37/40), and *emx1 *(11/13, 28/32) (Figure 1). However, four genes were not consistently upregulated by EBF3. The expression of *en-2 *(10/19) and *hoxd10 *(30/44) was downregulated, while the expression of *nr2f2 *was upregulated (19/73) in some embryos but downregulated (30/73) in others, and the expression of *wnt3a *(24/24) was not changed by EBF3 (data not shown). We therefore believe that these four genes are unlikely to be *in vivo *targets of EBF activity at this stage of early nervous system development, and we have excluded them from the experiments that follow. The fact that the expression levels of 10 genes among 14 candidates are upregulated by overexpression of EBF2 and EBF3 in the intact embryo supports the microarray data, and further shows that EBF2 and EBF3 activity are sufficient to drive expression of these candidate genes *in vivo*.

### EBF2 and EBF3 are required for the expression of candidate targets *in vivo*

**Figure 2:**
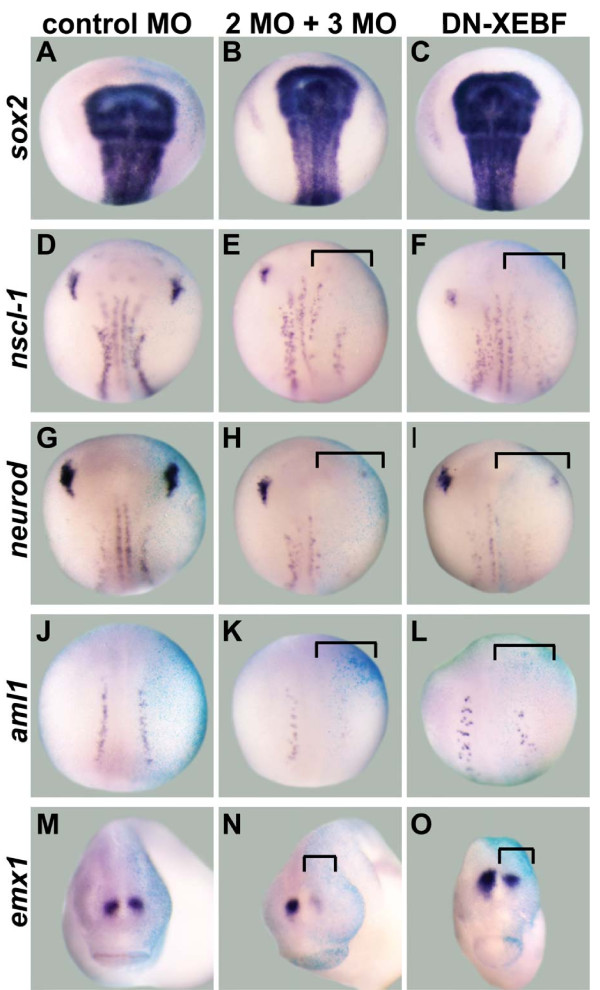
**Downregulation of transcription factor candidate target genes after knockdown of EBF2 and EBF3**. One cell of two-cell stage embryos was injected with either control MO, both EBF2 MO and EBF3 MO (2MO + 3MO), or dominant negative *Xenopus *EBF3 (DN-XEBF) mRNA. β-Galactosidase (β-gal) mRNA was co-injected as a marker of the injected side. In all panels the right side is the injected side, showing the light blue color of X-gal staining. **(A-C) **The expression of *sox2 *was not changed in all three conditions. **(D-O) **The expression of *nscl-1 *(E,F), *neurod *(H,I), *aml1 *(K,L), and *emx1 *(N,O) is downregulated by EBF2 MO and EBF3 MO, and by DN-XEBF (brackets), while control MO does not change their expression levels (D,G,J,M). Panels (A-L) show dorsal views of neurula stage embryos (stage 15/16), and (M-O) are anterior views of tail bud stage embryos (stages 25 to 28).

**Figure 3:**
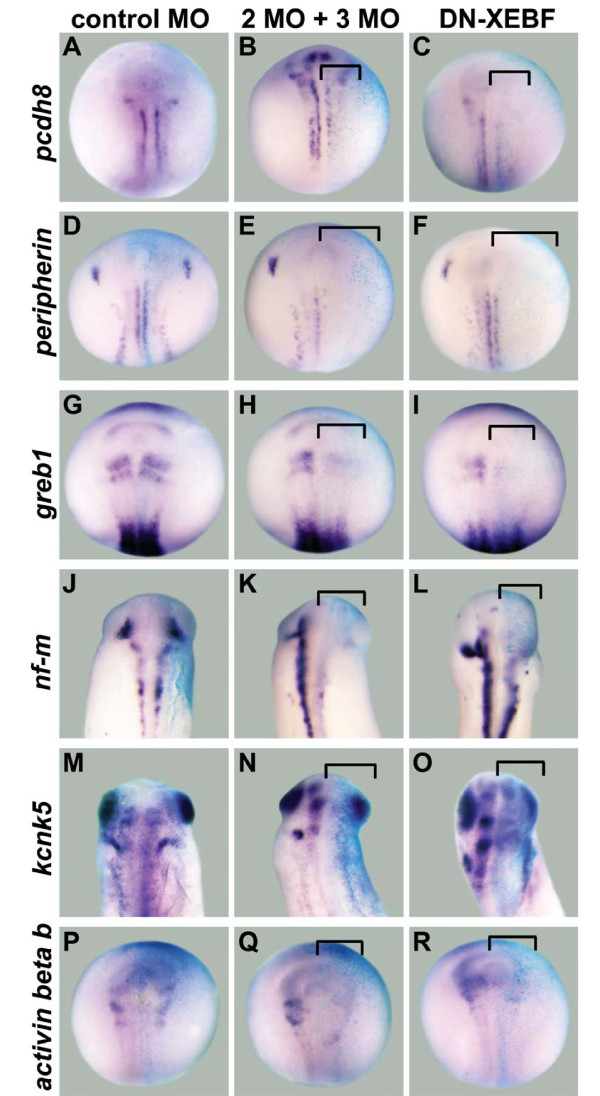
**Downregulation of non-transcription factor candidate target genes after knockdown of EBF2 and EBF3**. One cell of two-cell stage embryos was injected with either control MO, both EBF2 MO and EBF3 MO (2MO + 3MO), or dominant negative *Xenopus *EBF3 (DN-XEBF) mRNA. β-Galactosidase (β-gal) mRNA was co-injected as a marker of the injected side. In all panels the right side is the injected side, showing the light blue color of X-gal staining. **(A-R) **The expression of *pcdh8 *(B,C), *peripherin *(E,F), *greb1 *(H,I), *nf-m *(K,L), *kcnk5 *(N,O), and *activin beta b *(Q,R) is downregulated by EBF2 MO and EBF3 MO, and by DN-XEBF (brackets), while control MO does not change their expression levels (A,D,G,J,M,P). Panels (A-I) and (P-R) are neurula stage embryos (stage 15/16), and (J-O) are tail bud stage embryos (stages 25 to 28). All panels show dorsal views.

To determine if the expression of our identified candidate target genes is dependent on EBF2 and EBF3 *in vivo*, we examined the expression levels of candidate targets after knockdown of EBF2 and EBF3 expression using translation blocking antisense MOs. EBF2 MO and EBF3 MO were co-injected into one cell of two-cell stage embryos and the expression levels of endogenous candidate target genes were examined at the neurula stage (stage 15/16) or tailbud stage (stages 25 to 28), when expression of candidate target genes is apparent (Figures 2 and 3). We first confirmed that expression of the neural plate marker *sox2 *was not changed (10/10 embryos; Figure 2B), indicating that knockdown of EBF2 and EBF3 did not affect early global neuronal development in the early embryos. After co-injection of EBF2 MO and EBF3 MO, the expression of *nscl-1 *(10/11), *neurod *(13/15), *aml1 *(7/12), *emx1 *(12/15), *pcdh8 *(12/13), *peripherin *(11/11), *greb1 *(11/12), *nf-m *(14/16), *kcnk5 *(10/15) and *activin beta b *(5/10) were downregulated (Figures 2 and 3). Control MO did not change the expression levels of these genes (Figures 2 and 3). The expression of peripherin was partially rescued by co-injecting EBF2 MO and EBF3 MO together with mRNA for hGR-XEBF2, which does not have overlapping sequence with the MOs, and then treating with DEX from the gastrula stage (stage 11.5) to the neurula stage (stage 15/16) (Additional file 5). This control demonstrates that the EBF2 MO and EBF3 MO specifically block EBF activity.

To confirm the MO results, we generated a dominant negative *Xenopus *EBF3 construct (DN-XEBF). This DN-XEBF (amino acids 349 to 598) lacks the DNA binding domain in the amino-terminal region, but it has an intact dimerization domain [4,7,12]. Since EBF1, 2, and 3 can form homodimers or heterodimers *in vitro *[2,4,12], this DN-XEBF is predicted to block the function of both EBF2 and EBF3 by forming non-functional dimers. Similar to our MO data, injection of mRNA encoding DN-XEBF led to downregulation of the expression of *nscl-1 *(5/13 embryos), *neurod *(12/18), *aml1 *(6/14), *emx1 *(7/15), *pcdh8 *(8/18), *peripherin *(8/13), *greb1 *(8/14), *nf-m *(10/19), *kcnk5 *(7/19) and *activin beta b *(8/17) (Figures 2 and 3) while *sox2 *expression was not changed by DN-XEBF at the neurula stage (13/14) (Figure 2C). However, the level of downregulation of candidate target genes was weaker than that obtained by MO injection, perhaps because some endogenous EBF protein is able to form normal dimers even in the presence of DN-XEBF. In addition, a majority of embryos became bent toward the injected side at the tailbud stage because this side was smaller than the uninjected side (data not shown), suggesting changes in the development of other tissues. Taken together, these function-blocking experiments with MOs and DN-XEBF suggest that EBF2 and EBF3 are required for the expression of our neuronal candidate targets *in vivo*.

### Comparison of the expression patterns of EBF2, EBF3 and their candidate targets in the *Xenopus *nervous system

**Figure 4:**
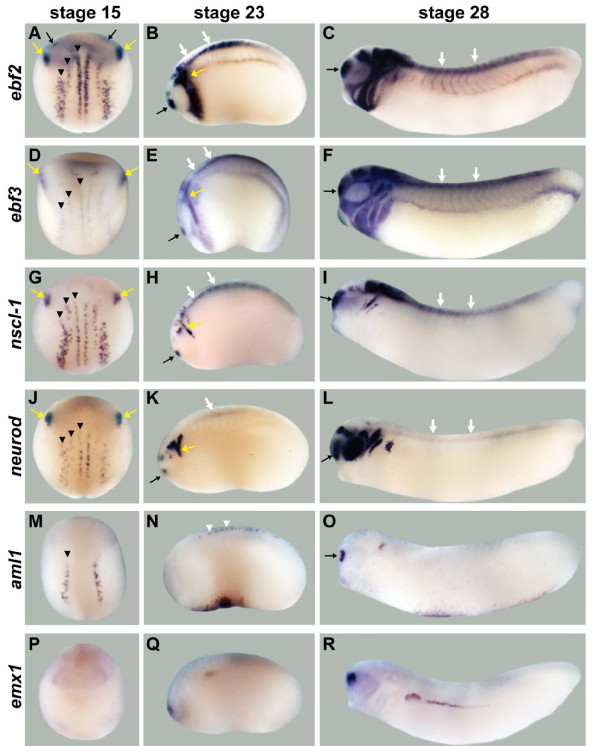
**Neuronal expression for *ebf *genes and transcription factor candidate target genes**. **(A-F) ***ebf2 *(A-C) and *ebf3 *(D-F) are expressed in multiple regions of the developing nervous system, including the trigeminal placodes (yellow arrows), olfactory placodes (black arrows), some domains in the brain, the spinal cord (white arrows), and neural crest derivatives like the branchial arches. **(G-L) ***nscl-1 *(G-I) and *neurod *(J-L) are expressed in the trigeminal placodes and three stripes of primary neurons in the neural plate (black arrowheads) at stage 15, and are strongly expressed in the trigeminal placodes, olfactory placodes, and spinal cord at stage 23. At stage 28, *nscl-1 *is expressed in the olfactory placodes, some domains in the midbrain/hindbrain, spinal cord and cranial ganglia IX and X. At stage 28, *neurod *is expressed in the olfactory placodes, retina, otic placodes, cranial ganglia, spinal cord and some domains in the brain. **(M-O) ***aml1 *is expressed in the lateral primary neuron stripe at stage 15, sensory neurons of the spinal cord (white arrowheads) at stage 23, and the olfactory placodes and otic placodes at stage 28. **(P-R) ***emx1 *is weakly expressed in the prospective forebrain region at stage 15. At stages 23 and 28, this gene is expressed in the dorsal forebrain region. Stage 15 embryos show dorsal views except **(P)** (anterior view). Stage 23 and 28 embryos show lateral views.

**Figure 5:**
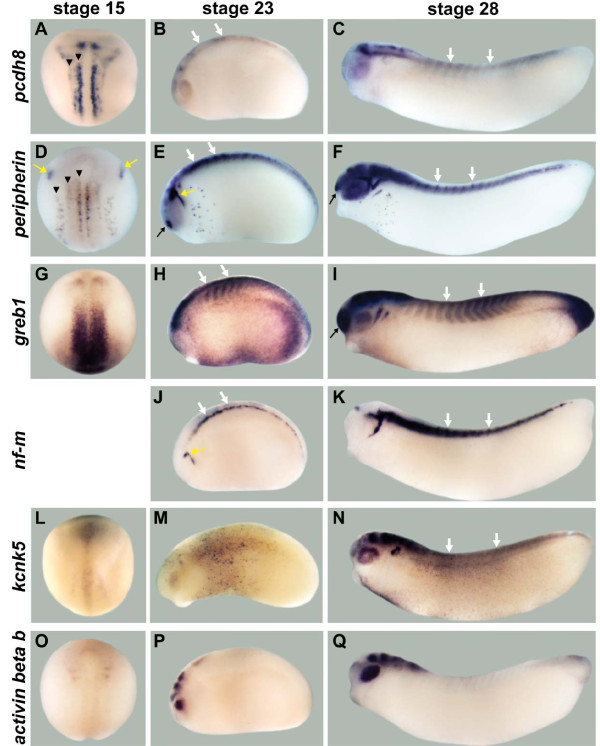
**Neuronal expression for non-transcription factor candidate target genes**. **(A-C) ***pcdh8 *is expressed in medial and intermediate stripes (arrowheads) of primary neurons, in one posterior stripe between the two stripes of primary neurons, and in the anterior domain of the neural plate at stage 15, and in the spinal cord (white arrows) and some domains in the brain at stages 23 and 28. **(D-F) ***peripherin *is expressed in the trigeminal placodes (yellow arrows) and three stripes of primary neurons (arrowheads) at stage 15, and in the trigeminal placodes, olfactory placodes (black arrow), spinal cord, retina and many domains in the brain at stages 23 and 28. **(G-I) ***greb1 *is expressed as a band in the prospective midbrain/hindbrain region at stage 15, and in the midbrain/hindbrain region and spinal cord at stage 23, and in the olfactory placodes, spinal cord, and many domains in the brain at stage 28. **(J,K) ***nf-m *is not expressed at stage 15, but at stages 23 and 28 it is expressed in the trigeminal placodes and spinal cord. **(L-N) ***kcnk5 *is weakly expressed from anterior to posterior along the dorsal midline, with stronger expression in the anterior end of the neural fold at stage 15. It is expressed in retina, otic placode, and several domains in the brain at stages 23 and 28, and in spinal cord at stage 28. **(O-Q) ***activin beta b *is expressed in two bands in the prospective midbrain/hindbrain region and diffusely throughout the anterior neural plate at stage 15, and it is expressed in the retina and some brain domains at stages 23 and 28. Stage 15 embryos show dorsal views, and stages 23 and 28 embryos show lateral views.

To determine if the functional relationships we identified above might be indicative of *in vivo *genetic relationships between *ebf *genes and candidate targets, and to determine if the candidate targets have expression patterns consistent with a role in neuronal development, we compared the expression domains of *ebf2 *and *ebf3 *with those of candidate target genes by WM-ISH at four different stages in early *Xenopus *embryos: stage 12.5 (data not shown), 15, 23 and 28 (Figures 4 and 5). We chose these stages because the expression of *ebf2 *is visible from stage 12.5, the expression of *ebf3 *is clearly visible at stage 15, and their expression continues beyond stage 28 [7,8]. *ebf2 *and *ebf3 *are expressed in very similar neuronal tissues (Figure 4A-F). At stage 15, both are expressed in the three stripes (medial, intermediate, and lateral) of primary neurons in the neural plate and trigeminal placodes. At stage 23, both are expressed in the trigeminal placodes, the olfactory placodes, spinal cord, and neural crest derivatives, including branchial arches. By stage 28, their expression expands to encompass much of the developing brain.

First, we compared the expression patterns of *ebf *genes and the candidate targets that are known transcription factors since a number of candidate target genes fell into this category (Figure 4). The expression patterns of both bHLH transcription factors, *nscl-1 *(Figure 4G-I) [40] and *neurod *(Figure 4J-L) [33], overlap strongly with those of *ebf2 *and *ebf3 *in the three stripes of primary neurons, trigeminal placodes, olfactory placodes and spinal cord. In *Xenopus *embryos, the neuronal expression of *aml1 *(Figure 4M-O) [42,43] is limited to sensory neurons, including the lateral stripe at stage 15, sensory neurons in the spinal cord at stage 23, and olfactory placode at stage 28, and this expression pattern overlaps with the expression of *ebf2 *and *ebf3*. The expression of *emx1 *(Figure 4P-R) [44] in the dorsal forebrain region at stages 23 and 28 overlaps with the expression of *ebf2 *and *ebf3*.

Second, we compared the expression patterns of *ebf *genes and the candidate targets that do not have transcriptional activity, and that were predicted to play a role in cell structure and neuronal function (Figure 5). The expression of *pcdh8 *(Figure 5A-C) does partially overlap with that of *ebf2 *and *ebf3 *in two stripes of primary neurons, spinal cord, midbrain, and hindbrain. The neuronal intermediate filament genes *peripherin *(Figure 5D-F) [51] and *nf-m *(Figure 5J,K) [52] show strongly overlapping expression patterns with *ebf2 *and *ebf3 *in the three stripes of primary neurons, trigeminal placodes, olfactory placodes and spinal cord from the earliest stages in which they are expressed. The expression patterns of *greb1 *(Figure 5G-I), *kcnk5 *(Figure 5L-N) and *activin beta b *(Figure 5O-Q) [50] do not overlap with those of *ebf2 *and *ebf3 *at stage 15, but partially overlap in some domains in the brain or the spinal cord at stages 23 or 28.

In summary, the candidate EBF target genes show complex patterns of expression during nervous system development with different degrees of overlap with the EBF factors. For example, some were highly overlapping, *nscl-1*, *neurod*, *aml1*, *peripherin*, and *nf-m*, while others showed more limited overlap, such as *emx1*, *pcdh8*, *greb1*, *kcnk5*, and *activin beta b*. The genes *pcdh8*, *greb1*, and *kcnk5 *have not previously been described during *Xenopus *neuronal development.

### Identification of direct candidate targets for EBF3 in animal caps by RT-QPCR

**Figure 6:**
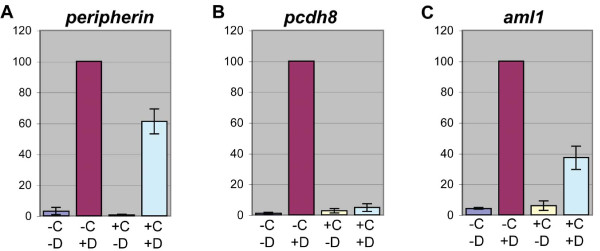
**The identification of direct and indirect candidate targets of EBF3 by RT-QPCR**. hGR-XEBF3 mRNA and Noggin mRNA were injected into one-cell stage embryos, and animal caps were collected at the blastula stage (stage 9). The animal caps were divided into four groups, based on CHX and DEX treatment: -C-D, -C+D, +C-D, and +C+D. After a 3.5-hour incubation with CHX and/or a 3-hour incubation with DEX, total RNA was isolated from each animal cap group. RT-QPCR was conducted with the isolated total RNA. The expression level was normalized with the expression level of *histone h4 *and then normalized to the expression level of -C+D, for each gene, at 100 arbitrary units. **(A-C) **The expression levels of all candidate target genes in controls (-C-D and +C-D) are very low compared to the DEX-treated condition of -C+D. (A) The expression level of *peripherin *in +C+D (61%) is only partially reduced compared to -C+D, indicating that the majority of its expression is controlled by EBF3 directly. (B) The expression level of *pcdh8 *in +C+D (5%) is much lower than in -C+D and is similar to the levels of the control conditions, indicating that it is an indirect candidate target. (C) The expression level of *aml1 *in +C+D (37%) is lower than the expression level in -C+D but higher than levels of the control conditions, indicating that its expression is partially controlled by EBF3 directly. Error bars represent standard error of the mean. The results for the remaining candidate target genes are shown in Additional file 6. N = 3 replicates, 20 to 30 animal caps per condition.

To better understand the transcriptional interaction between EBF3 and its candidate target genes, we sought to identify which genes are direct transcriptional candidate targets and which are indirect transcriptional candidate targets. We used an approach similar to the microarray analysis, with DEX treatment of animal caps to drive activation of hGR-XEBF3, but we added CHX to block protein synthesis, so that only direct EBF3 candidate targets should be transcribed. Animal caps were collected at stage 9 after injection of hGR-XEBF3, and divided into four groups: untreated control (-C-D), DEX alone (-C+D), CHX alone (+C-D), and both CHX and DEX (+C+D). All animal caps were collected after a 3.5-hour incubation. CHX treatment lasted the entire 3.5 hours, while DEX treatment started after a 30-minute delay to allow time for CHX to take effect. The expression level of each candidate target was examined by RT-QPCR (Table 1 and Figure 6; Additional file 6). We normalized the expression level of candidate target genes with that of *histone h4*, and for Figure 6 and Additional file 6 we set the normalized expression level in the condition of -C+D to 100% (see Materials and methods).

After treatment with both CHX and DEX, candidate targets that had expression levels of less than 10% of the level in animal caps treated with DEX alone (or candidates that had expression levels similar to untreated controls, even if that was greater than 10% of the DEX alone condition) were considered to be indirect candidate targets. These indirect targets are *pcdh8*, *kcnk5*, *activin beta b*, *neurod *and *greb1 *(Figure 6B; Additional file 6D-G). Candidate target genes with expression levels of greater than 50% of the levels in animal caps treated with DEX alone are candidate targets for which the majority of their expression is directly controlled by EBF3. These include *peripherin*, *emx1 *and *nf-m *(Figure 6A; Additional file 6AB). Finally, there are genes with expression levels between 10 and 50% of the level in animal caps treated with DEX alone. These genes, including *aml1 *and *nscl-1*, likely have some expression that is under direct regulation by EBF3 (Figure 6C; Additional file 6C), suggesting that their expression is controlled not only by EBF3 directly but also through other targets of EBF3.

## Discussion

To better understand the range of activities that are driven by EBF transcription factors during neural development, we used a systematic approach to identify candidate target genes of EBF activity in *Xenopus*. In this study, we emphasize candidate targets that were highly upregulated by EBF activity by microarray analysis and with potential functions in neuronal development. Our microarray screen for targets of EBF transcriptional activity revealed many genes that were previously not known to be targets of EBF activity. Ten of the genes showed upregulation after EBF overexpression, and downregulation after EBF knockdown in neurula or tailbud stage embryos, demonstrating their *in vivo *dependence on EBF activity. In addition, all ten of these genes partially overlap in their expression with *ebf *genes, consistent with regulation by EBF proteins during neural development. Since some of these genes are direct targets with partial dependency on EBF activity, others are indirect candidate targets, and most have only partially overlapping expression with *ebf *genes, it is clear that EBF proteins are part of a more complex transcriptional regulatory network involved in driving their expression. The candidate target genes we identified by microarray analysis, but did not characterize further, also have the potential to reveal involvement of EBF proteins in additional activities, and this list of genes should aid future research into EBF functions.

Several previous studies, including EBF gain- and loss-of-function studies in *Drosophila*, *C. elegans*, *ebf *mutant mice, chicks, *Xenopus*, and in cell lines, have revealed a number of neuronal genes that are regulated by EBF transcriptional activity [2,7,8,15-17,19,53-56]. With the exception of *nf-m*, the list of candidate EBF target genes that we selected for analysis does not include these previously described EBF-regulated genes. However, among the additional genes from the microarray that are upregulated by EBF activity we found *lhx9 *and *lmo4*, which are homologous to *apterous*, a known target of Collier in *Drosophila *[17]. It is likely that we did not recognize the presence of other known EBF target genes in our array results due to the incomplete annotation of the *Xenopus *microarray. Another explanation could be that expression of these targets is driven by EBF in the more differentiated environments of each independent experiment, but not in the relatively naïve environment of *Xenopus *animal caps. Future improvements in *Xenopus *annotation should make it possible to help distinguish between these two explanations.

We find in our *Xenopus *EBF screens comparable numbers of strongly downregulated genes and strongly upregulated genes. This parallels the important roles shown for EBF proteins functioning as transcriptional repressors to help determine cell fate in cell types such as B cells in mice [37,57,58] and ASI chemosensory neurons in *C. elegans *[55]. As part of a future study, it will be interesting to see the extent to which our candidate downregulated genes are related to cell fate.

EBF proteins are involved in the development of multiple lineages, including B cells and adipocytes. Interestingly, in our array analysis we found genes with known roles in non-neuronal tissue types like *myod*, which regulates muscle development, or *lmo2 *and *hex*, which are involved in leukocyte development. These genes were upregulated even after animal caps were neuralized with Noggin. Thus, in the relatively naïve environment of animal cap ectoderm it may be permissive for EBF target genes from multiple lineages to be expressed. EBF proteins have been proposed to function as pioneer factors for B cell genes in hematopoietic lineages [37]. Therefore, EBF factors may alter chromatin to permit genes involved in lineage specification and differentiation to be expressed. There have been screens performed to identify targets of EBF proteins in non-neuronal cell types such as B cells and adipocytes [37,59,60]. A detailed comparison of our array results with these studies is difficult since limited annotation of the *Xenopus *arrays precludes systematic cross-species comparisons.

### EBF regulation of multiple transcription factor genes suggests involvement in extensive transcriptional networks for neuronal development

Our finding that several transcription factors are among the strongest candidate targets of EBF activity expands the potential means by which EBF activity could exert its many effects on neuronal development, and also suggests some interesting new potential functions. The bHLH transcription factor NSCL-1 can drive expression of the proneural bHLH transcription factor NGNR-1, which is important for neuronal cell commitment in *Xenopus *embryos [40,61]. In chick and mouse, NSCL-1 can promote neuronal cell differentiation, and migration of cellular populations, including GnRH-1 neurons [62-64]. Interestingly, *Ebf2 *knockout mice show a migration defect of GnRH-1 neurons [21]. We find that the expression of *nscl-1 *is partially under the direct control of EBF activity, and that the expression patterns of *nscl-1 *and *ebf *genes strongly overlap. This suggests that EBF activity may act through NSCL-1 to regulate neuronal cell commitment, differentiation or migration.

The well-known functions of the proneural bHLH transcription factor NeuroD in multiple species show that it is involved primarily in differentiation, but also acts to regulate cell fate, cell migration and cell survival [33,65-68]. This study and previous studies show that *neurod *expression is very similar to that of *ebf *genes [7,8,33]. Previous studies showed that *neurod *is both downstream of EBF2 and upstream of *ebf2 *and *ebf3 *in *Xenopus *embryos [7,8,28,65]. Our present data suggest that *neurod *is also an indirect candidate target of EBF3. Together, these results support and expand the concept of multiple transcriptional interactions between EBF proteins and NeuroD [7,8,28,65].

AML1, a runt related transcription factor, is known to be expressed in neurons, including cortical progenitors, olfactory receptor progenitors and neurons in the dorsal root ganglia and to be involved in differentiation and cell type specification of several types of sensory and motor neurons, including neurons in the dorsal root ganglia [42,69-71]. Interestingly, AML1 is known to cooperate with EBF proteins in B cell development [72]. We find that *aml1 *is partially under the direct control of EBF activity, and that the expression patterns of *aml1 *and *ebf *genes overlap strongly in the nervous system. Thus, AML1 and EBF proteins may also act cooperatively in promoting neuronal differentiation.

In multiple species, the homeobox transcription factor Emx1 is strongly expressed in the developing forebrain, and the EMX1 protein is present in the axons of the olfactory neurons [44,73,74]. Compared to *Emx2 *knockout mice, *Emx1 *knockout mice show only minor defects in brain development [75-77]. However, *Emx1 *and *Emx2 *double mutant mice show more severe defects than *Emx2 *knockout mice, including defects of neuronal differentiation and thalamocortical pathfinding [78], similar to those found in *Ebf1 *knockout mice [16]. Since we find *emx1 *to be a strong, direct candidate target of EBF proteins, and *emx1 *and *ebf *genes are both strongly expressed in the forebrain, EBF proteins may control cell differentiation and axon growth in part by driving expression of *emx1*.

### EBF proteins drive expression of candidate targets involved in multiple aspects of neuronal differentiation

The candidate targets that are not transcription factors illuminate some of the ways that EBF activity could help regulate late steps of neuronal differentiation and neuronal function. Peripherin and NF-M are important components of neuronal intermediate filaments, which help to form the cytoskeleton in the cell body and neurites of neurons [79-82]. We find that the majority of expression of *peripherin *and *nf-m *is controlled directly by EBF3, and that their expression strongly overlaps with that of *ebf *genes. These discoveries correlate with previous evidence showing axonal pathfinding defects of thalamocortical and olfactory receptor neurons in *Ebf *null mice, pathfinding defects of motor neurons in *C. elegans *UNC-3 mutants, and problems with dendritic arborization in *Drosophila *Collier mutants [11,16,19,20,53,54,83]. These correlations both support a role for EBF proteins in axon growth or stability and provide a potential additional route for exploration of how EBF proteins can affect this important process.

PCDH8 is a transmembrane calcium-dependant adhesion molecule. The rat homolog Arcadlin affects the number of dendritic spines in cultured hippocampal neurons [84] and is required for activity-induced long-term potentiation [85]. We find that EBF proteins positively regulate the expression of a gene that is likely the *Xenopus pcdh8 *homolog (based on sequence similarity and similar range of gene expression with mouse *Pcdh8 *in midbrain, hindbrain and spinal cord [86]). We show that *pcdh8 *is an indirect candidate target of EBF activity, and that *pcdh8 *and *ebf *expression patterns overlap in the brain and spinal cord, suggesting that EBF proteins may be involved in synaptic plasticity by controlling the expression of *pcdh8*, which would be a new function for EBF proteins in the nervous system.

KCNK5 is a K+ channel that is sensitive to extracellular pH, and in rat kidney cells it functions to stabilize bicarbonate transport and control cell volume [49,87,88]. It appears to be involved in maintaining the membrane potential of chemoreceptor cells in mouse brainstem [89]. We find that *Xenopus kcnk5 *is indirectly upregulated by EBF activity. In addition, we find overlap between *ebf *and *kcnk5 *gene expression in the midbrain and hindbrain at the tailbud stage. Regulation of this gene represents a previously unknown function for EBF transcriptional activity.

Activin beta B forms homodimers, or heterodimers with Activin beta A. Activins are ligands of the TGF-beta superfamily, which are involved in differentiation in tissues from many systems, including the reproductive system [90-93]. Activin beta B is expressed in the developing brain and retina ([50,94,95] and our data), but its function in neuronal development is not yet clear. Our study shows that the *activin beta b *gene is likely an indirect candidate target of EBF proteins, and that its expression precedes that of *ebf *genes in midbrain, hindbrain and retina. These results suggest that EBF activity may maintain the expression of *activin beta b *instead of initiating its expression.

The protein GREB1 is thought to be involved in the estrogen-induced growth of breast cancer cells [96,97], but its function is not known, and its expression pattern in animal development has not been previously described. We find that *greb1 *is expressed in several tissues, including neurons and muscle cells, during *Xenopus *development. Overlapping expression with *ebf *genes is limited to the spinal cord and a few brain regions at tailbud stages, and we find that the expression of *greb1 *is controlled by EBF proteins indirectly. Our findings demonstrate a potential relationship between these genes and a possible role for GREB1 in neuronal development.

### Candidate targets that overlap most with *ebf *genes tend to be direct targets

Interestingly, the candidate targets having direct dependency on EBF3 for their expression, based on our CHX experiments (Figure 6; Additional file 6), tended to have the most overlap with *ebf *genes in their WM-ISH expression patterns (Figures 4 and 5). These included the genes that code for axonal structural proteins (*peripherin *and *nf-m*) and two that code for transcription factors (*aml1 *and *nscl-1*). In contrast, the genes that appear to be indirect candidate targets tended to be those that have the least overlap with *ebf *genes in their expression patterns. These included *pcdh8*, *kcnk5*, *activin beta b*, and *greb1*. An exception to this rule was *neurod*, which has extensive overlap with *ebf *genes, but is an indirect target. This could reflect the fact that *neurod *is also upstream of *ebf *genes [7,8,28,65]. These results strengthen the conclusion that *in vivo *expression of the candidate target genes determined to have direct dependency on EBF3 transcriptional activity is likely to be heavily dependent on EBF activity.

### EBF proteins function in cell commitment and differentiation in the development of multiple lineages

EBF proteins have been implicated in the specification, commitment, and differentiation of specific cell types by regulating the expression of both genes in transcriptional regulatory networks as well as genes involved in the functional activities of a cell type, consistent with what we found in our analysis. For example, EBF1 is known to participate in B cell specification, commitment, and differentiation by inducing the expression of transcription factors like E2A (Tcf3) and Pax5, and non-transcription factors like CD79a (mb-1) and VpreB [37,60,98,99]. EBF proteins similarly participate in adipocyte differentiation, by inducing the expression of both types of genes [59,100,101].

In B cells EBF1 acts cooperatively with other transcription factors, including AML1 (RUNX1), E2A, Pax5, and Foxo1, by driving expression of overlapping sets of target genes ([102] and reviewed in [36,103-105]). Interestingly, these transcription factors are themselves targets of EBF1 in B cells. Similar mechanisms are found during neuronal subtype development in the *Drosophila *ventral nerve cord [17]. The fact that AML1 is also one of our candidate targets, and that most of our transcription factor candidate targets have extensive and early co-expression with *ebf2 *and *ebf3*, suggests that EBF proteins may act cooperatively with their transcription factor targets for the expression of some genes in *Xenopus *neuronal development. Cooperative regulation of gene expression could be part of positive feedback mechanisms that solidify cell commitment choices and differentiated states.

### EBF2 and EBF3 appear to share most candidate targets during early *Xenopus *development

In the developing mouse nervous system, there is evidence for areas of overlap in the functions of different members of the EBF family. For example, *Ebf2 *and *Ebf3 *knockout mice have similar phenotypes for olfactory axon growth [20], and *Ebf1 *null mice do not show severe defects in the brain regions expressing multiple EBF family members concomitantly [1,2,5,16,98]. However, there is also evidence for distinct functions of different members, including the fact that *Ebf1 *is the only member expressed in the embryonic striatum and *Ebf1 *null mice show defective neuronal cell differentiation in that region [1,16]. In addition, *Ebf2 *null mice show defective migration of GnRH-1 neurons even though *Ebf1 *is also expressed in those neurons [21,106]. In *Xenopus*, the previously known functions of EBF2 and EBF3 are very similar during early development [7,8]. They are both important for neuronal differentiation, including control of the expression of the neuronal specific markers N-tubulin, N-CAM, and NF-M. Supporting the similarity of the roles of these two genes, we find that the ten candidate targets of EBF3 that were upregulated by hGR-XEBF3 in *Xenopus *animal caps and *in vivo *could also be upregulated by hGR-XEBF2 *in vivo*. Although there is interesting evidence for some differences in expression patterns and functions of EBF2 and EBF3 [7,8], our results support the idea that EBF2 and EBF3 have largely redundant functions at the transcriptional level.

## Conclusions

We have found multiple EBF candidate targets using a systematic approach in *Xenopus *embryos. The expression patterns of direct candidate targets of EBF3 have strong overlap with *ebf *gene expression, while candidate targets having largely indirect dependency on EBF3 are expressed in less overlapping patterns, suggesting more complex modes of regulation. The novel candidate target genes suggest new potential routes for EBF transcription factors to carry out their previously known functions of neuronal cell commitment, differentiation, neurite formation and migration, and also suggest some new potential functions of EBF activity.

## Abbreviations

bHLH: basic helix-loop-helix; CHX: cycloheximide; COE: Collier/Olf/Ebf; DEX: dexamethasone; EBF: early B cell factor; GEO: Gene Expression Omnibus; GnRH: gonadotropin releasing hormone; hGR: human glucocorticoid receptor; MO: morpholino; *nf-m*: *neurofilament-m*; O/E: Olf/Ebf; RT-PCR: reverse transcriptase PCR; RT-QPCR: real-time quantitative PCR; TGF: transforming growth factor; WM-ISH: whole mount *in situ *hybridization.

## Competing interests

The authors declare that they have no competing interests.

## Authors' contributions

YSG participated in the design of these experiments, performed the experiments, analyzed most of the data, and wrote the manuscript. MLV guided the design of the experiments and aided the writing and data analysis. Both authors approved the final manuscript.

## Supplementary Material

Additional file 1 **Hormone inducible constructs and scheme of microarray screens. (A) **Schematic diagram of the hormone inducible constructs used in the microarrays. **(B) **Schematic representation of the microarray screen comparing transcripts in DEX-treated animal caps (with activated hGR-XEBF3) and untreated animal caps (without activated hGR-XEBF3), as well as the control array to analyze the effects of DEX treatment. The scheme shown represents the arrays performed in the presence of Noggin mRNA. Click here for file

Additional file 2 **Primer sequences used for RT-QPCR**. Click here for file

Additional file 3 **Microarray results table showing genes up- or downregulated more than two-fold by EBF3 activity in the presence of Noggin**. Click here for file

Additional file 4 **Microarray results table showing genes up- or downregulated more than two-fold by EBF3 activity in the absence of Noggin**. Click here for file

Additional file 5 **Expression of *peripherin *in embryos treated with EBF2 MO and EBF3 MO can be rescued by co-injection of hGR-XEBF2**. One cell of two-cell stage embryos was injected with EBF2 MO, EBF3 MO and mRNA encoding hGR-XEBF2, followed by DEX treatment (or no treatment in controls) from the late gastrula stage (stage 11.5) to the neurula stage (stage 15/16). β-Galactosidase (β-gal) mRNA was co-injected as a marker of the injected side. In both panels the right side is the injected side (brackets). In control embryos (without DEX treatment) *peripherin *expression was downregulated either strongly (3/7, shown in (A)) or weakly (4/7, not shown) compared to the uninjected side due to the MO effect. In the majority of DEX-treated embryos *peripherin *expression was either rescued (16/34, shown in (B)), or only weakly downregulated (17/34, not shown). Both panels show dorsal views. Click here for file

Additional file 6 **Additional identification of direct and indirect candidate targets of EBF3 by RT-QPCR**. Expression levels for the remaining candidate target genes tested by RT-QPCR after CHX and DEX treatment (those not shown in Figure 6). **(A,B) **The expression levels of *emx1 *(88%) and *nf-m *(56%) in +C+D are slightly lower than in -C+D, indicating that the majority of their expression is controlled by EBF3 directly. **(C) **The expression level of *nscl-1 *(46%) in +C+D is lower than in -C+D but higher than that in the two controls, indicating that its expression is under partial direct control of EBF3. **(D-G) **The expression levels of *kcnk5 *(20%), *activin beta b *(10%), *neurod *(8%) and *greb1 *(1%) in +C+D are similar to control levels (D) or much lower (less than 10%) than in -C+D (E-G). N = 20 to 30 animal caps per condition. Click here for file
