# Supplementary figures and images for: EBF factors drive expression of multiple classes of target genes governing neuronal development
Source: Neural Dev. 2011 Apr 30;6:19. doi: 10.1186/1749-8104-6-19 (PMC3113313; doi:10.1186/1749-8104-6-19)

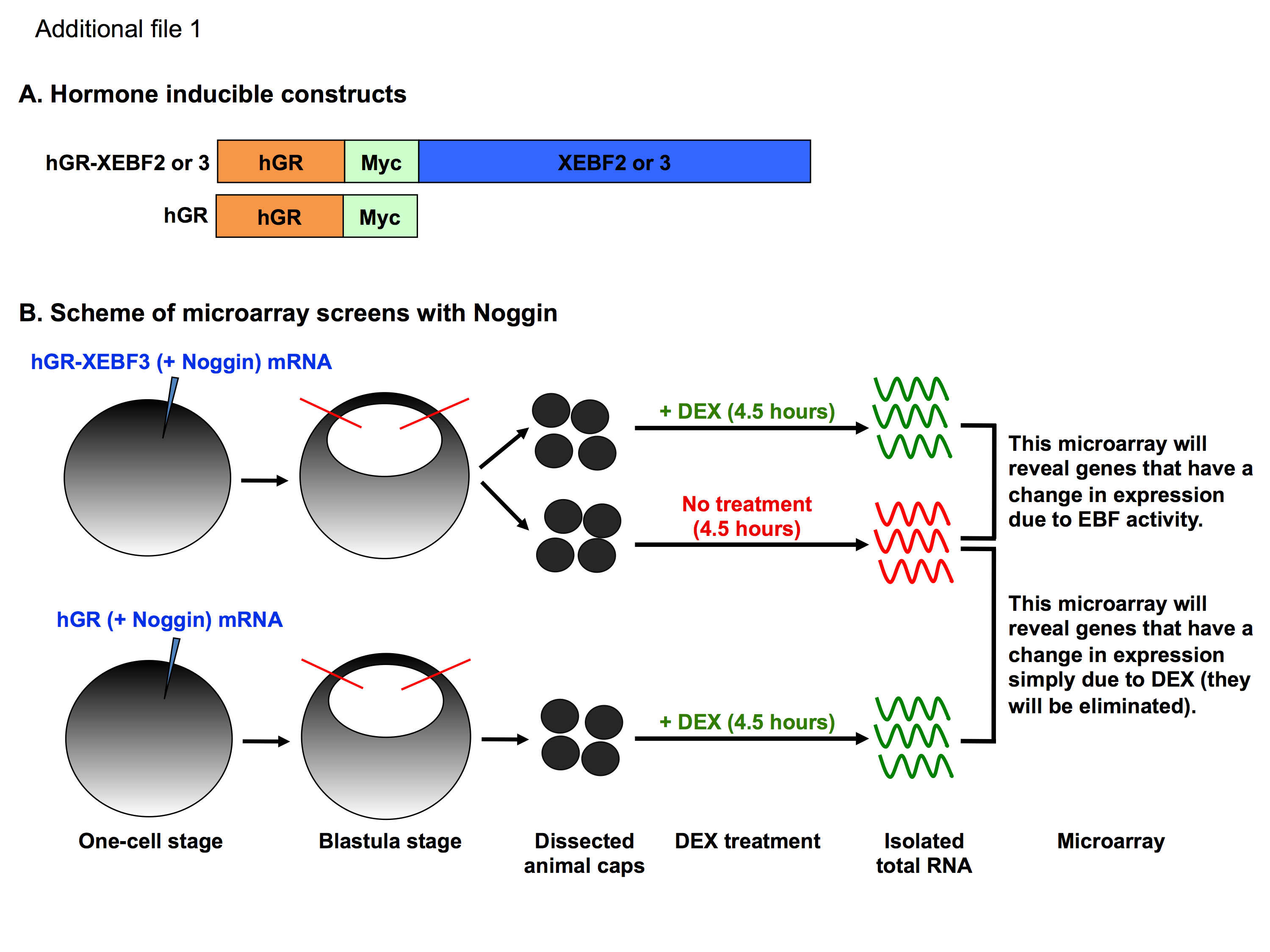

Supplement: Additional file 1 — Hormone inducible constructs and scheme of microarray screens. (A) Schematic diagram of the hormone inducible constructs used in the microarrays. (B) Schematic representation of the microarray screen comparing transcripts in DEX-treated animal caps (with activated hGR-XEBF3) and untreated animal caps (without activated hGR-XEBF3), as well as the control array to analyze the effects of DEX treatment. The scheme shown represents the arrays performed in the presence of Noggin mRNA. [file 1749-8104-6-19-S1.PNG]

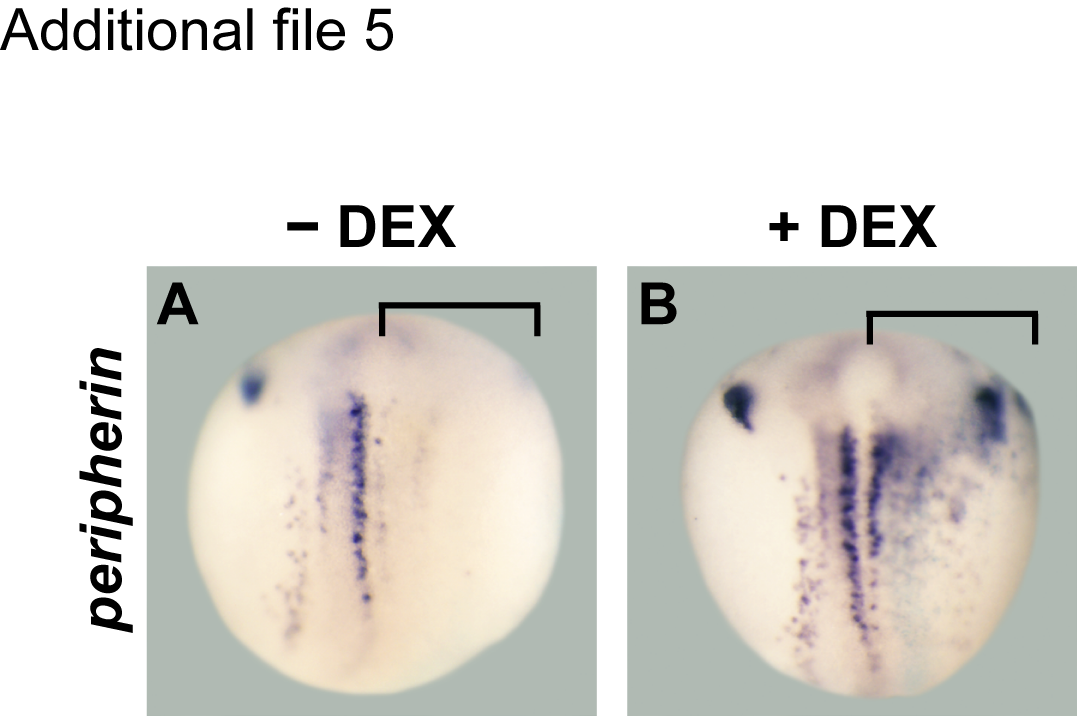

Supplement: Additional file 5 — Expression of peripherin in embryos treated with EBF2 MO and EBF3 MO can be rescued by co-injection of hGR-XEBF2. One cell of two-cell stage embryos was injected with EBF2 MO, EBF3 MO and mRNA encoding hGR-XEBF2, followed by DEX treatment (or no treatment in controls) from the late gastrula stage (stage 11.5) to the neurula stage (stage 15/16). β-Galactosidase (β-gal) mRNA was co-injected as a marker of the injected side. In both panels the right side is the injected side (brackets). In control embryos (without DEX treatment) peripherin expression was downregulated either strongly (3/7, shown in (A)) or weakly (4/7, not shown) compared to the uninjected side due to the MO effect. In the majority of DEX-treated embryos peripherin expression was either rescued (16/34, shown in (B)), or only weakly downregulated (17/34, not shown). Both panels show dorsal views. [file 1749-8104-6-19-S5.PNG]

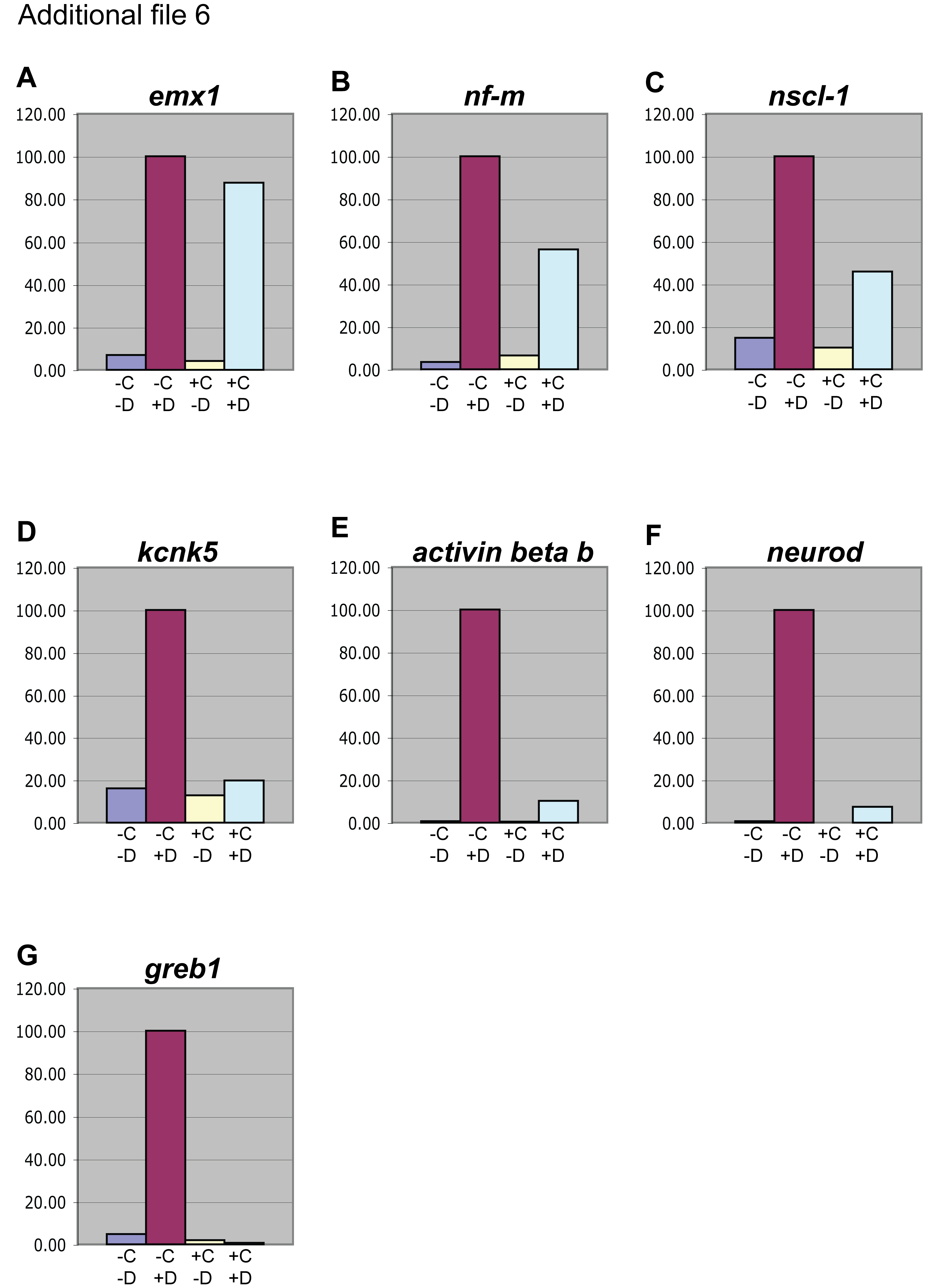

Supplement: Additional file 6 — Additional identification of direct and indirect candidate targets of EBF3 by RT-QPCR. Expression levels for the remaining candidate target genes tested by RT-QPCR after CHX and DEX treatment (those not shown in Figure 6). (A,B) The expression levels of emx1 (88%) and nf-m (56%) in +C+D are slightly lower than in -C+D, indicating that the majority of their expression is controlled by EBF3 directly. (C) The expression level of nscl-1 (46%) in +C+D is lower than in -C+D but higher than that in the two controls, indicating that its expression is under partial direct control of EBF3. (D-G) The expression levels of kcnk5 (20%), activin beta b (10%), neurod (8%) and greb1 (1%) in +C+D are similar to control levels (D) or much lower (less than 10%) than in -C+D (E-G). N = 20 to 30 animal caps per condition. [file 1749-8104-6-19-S6.PNG]
