# Supplementary material for: EBF factors drive expression of multiple classes of target genes governing neuronal development
Source: Neural Dev. 2011 Apr 30;6:19. doi: 10.1186/1749-8104-6-19 (PMC3113313; doi:10.1186/1749-8104-6-19)
Supplement: Additional file 2 — Primer sequences used for RT-QPCR. [file 1749-8104-6-19-S2.DOC]

Additional file 2

| **Name** | **Sequence** |
| --- | --- |
| *pcdh8* forward | 5’- AGGACAGCGGCAAAGGTGAC -3’ |
| *pcdh8* reverse | 5’- GGCGGGGAGAGCAGATTTAG -3’ |
| *peripherin* forward | 5’- CCAAGCAAAGTCCAAAGAGCC -3’ |
| *peripherin* reverse | 5’- GGTTGTGCCTGAACGGTCAC -3’ |
| *greb1* forward | 5’- TGACAAAAGGTTGGGCAGGG -3’ |
| *greb1* reverse | 5’- AGGAAAACTATCGGCGGCTG -3’ |
| *nf-m* forward | 5’- GAACAGGTACGCCAAGCTGACTG -3’ |
| *nf-m* reverse | 5’- GCAGCAATTTCTATATCCAGAG -3’ |
| *kcnk5* forward | 5’- CGGGTTTGGAGACTATGTGGC -3’ |
| *kcnk5* reverse | 5’- ATCCTTTGGGTTGGTCATTGG -3’ |
| *nscl-1* forward | 5’- TTCCATTGCTCCGTCAAGTTTC -3’ |
| *nscl-1* reverse | 5’- GCCCATCGTGTCCATTGTTTTC -3’ |
| *neurod* forward | 5’- CCAGAAACCCAAAAGACGAGG -3’ |
| *neurod* reverse | 5’- ATGCGACGGCACATCCTGAC -3’ |
| *aml1* forward | 5’- AACCAACCCAATCCAAGCAGTAG -3’ |
| *aml1* reverse | 5’- CAGCAACCTGTCCTGTATGTTCC -3’ |
| *activin beta b* forward | 5’- ATGATTGTGGACGAGTGCGG -3’ |
| *activin beta b* reverse | 5’- TGCTTCTATCCCTTTGCCAGG -3’ |
| *emx1* forward | 5’- CGCTCCATCTACAACAACCCG -3’ |
| *emx1* reverse | 5’- ATGTCGCTGCCTTGAAATCTG -3’ |
| *histone h4* forward | 5’- TGCGGGATAACATTCAGGGC -3’ |
| *histone h4* reverse | 5’- CGGTCTTCCTCTTGGCGTG -3’ |
